# Fruit and vegetable consumption and risk of cholecystectomy: a prospective cohort study of women and men

**DOI:** 10.1007/s00394-016-1298-6

**Published:** 2016-08-20

**Authors:** Caroline Nordenvall, Viktor Oskarsson, Alicja Wolk

**Affiliations:** 1 Department of Molecular Medicine and Surgery, Karolinska Institutet, Stockholm, 171 76 Sweden; 2 Center for Digestive Diseases, Department of Coloproctology, Karolinska University Hospital, 171 76 Stockholm, Sweden; 3 Institute of Environmental Medicine, Karolinska Institutet, 171 77 Stockholm, Sweden

**Keywords:** Fruit consumption, Vegetable consumption, Gallbladder disease, Cohort study

## Abstract

**Purpose:**

Epidemiologic data on whether consumption of fruit and vegetables (FVs) decreases the risk of gallstone disease are sparse. Therefore, we examined the association between FV consumption and the 14-year risk of symptomatic gallstone disease (defined as occurrence of cholecystectomy) in a large group of middle-aged and elderly persons.

**Methods:**

Data from two population-based cohorts were used, which included 74,554 men and women (born 1914–1952). Participants filled in a food frequency questionnaire in the late fall of 1997 and were followed up for cholecystectomy between 1998 and 2011 via linkage to the Swedish Patient Register. Cox regression models were used to obtain hazard ratios (HRs).

**Results:**

During 939,715 person-years of follow-up, 2120 participants underwent a cholecystectomy (1120 women and 1000 men). An inverse association between FV consumption and risk of cholecystectomy was observed in age- and sex-adjusted analyses (*P*_trend_ = .036) but not in confounder-adjusted analyses (*P*_trend_ = .43). The multivariable-adjusted HR was 0.95 (95 % CI 0.83–1.08) for the highest compared with the lowest sex-specific quartile of FV consumption. There was no evidence of interactions with age (*P* = .25) or sex (*P* = .72) in analyses pooled by sex. However, an age-by-FV consumption interaction was observed in separate analyses of women (*P* = .010), with decreased HRs of cholecystectomy for ages up to 60 years.

**Conclusions:**

This study supports an inverse association between FV consumption and risk cholecystectomy in women, although the association was restricted to women aged 48–60 years. In contrast, the study does not support an association in men.

**Electronic supplementary material:**

The online version of this article (doi:10.1007/s00394-016-1298-6) contains supplementary material, which is available to authorized users.

## Introduction

In order to improve health and longevity, a high consumption of fruit and vegetables (FVs) is recommended as part of a healthy diet [1]. While a variety of chronic diseases have been studied with regard to FV consumption up to a meta-analysis level, such as type 2 diabetes [2], cardiovascular disease [3], and cancer [4–6]; its association with gallstone disease has seldom been studied, particularly in large prospective cohort studies. In the only such study, which was performed in the Nurses’ Health Study (NHS), it was observed that women who were high consumers of FVs had a decreased risk of cholecystectomy (used as a proxy for symptomatic gallstone disease) [7]. In other studies (i.e., in small cohort, case–control, and cross-sectional studies), both inverse [8, 9] and null [10–14] associations have been observed between FV consumption and risk of gallstone disease (defined as the presence of gallstones on ultrasound and/or occurrence of cholecystectomy).

To investigate whether the findings from the NHS were possible to reproduce in a cohort of European women, as well as to provide large prospective data on men for the first time, we examined the association between FV consumption and the 14-year risk of cholecystectomy in two prospective cohorts of middle-aged and elderly persons.

## Methods

### Cohorts

The Swedish Mammography Cohort is a population-based cohort of 66,651 women, aged 40–75 years at recruitment in 1987–1990. All women answered a baseline questionnaire with questions on diet and lifestyle characteristics (i.e., marital status, education, height, and weight) and 39,227 of them (then aged 48–83 years) answered a second questionnaire in 1997 with expanded questions on diet and lifestyle characteristics as well as with additional questions on medication use, supplement use, and medical history. The Cohort of Swedish Men is a population-based cohort of 48,850 men, aged 45–79 years at recruitment in 1997, who answered a questionnaire that was identical to the second questionnaire in the Swedish Mammography Cohort. Details on the cohorts can be viewed at www.ki.se/en/imm/unit-of-nutritional-epidemiology.

This analysis was restricted to women and men who completed the questionnaire in 1997 (because the first female questionnaire did not contain questions on a number of important co-variables, such as cigarette smoking, physical activity, and use of postmenopausal hormones; *n* = 88,077). At baseline, we excluded those with incorrect personal identity numbers (*n* = 540), implausible energy intakes (±3 standard deviations of the sex-specific log-transformed mean; *n* = 1090), and histories of cancer or cholecystectomy (*n* = 10,413) as well as those with missing data on fruit consumption or vegetable consumption (*n* = 981). In addition, we excluded participants who developed cancer in the duodenum, liver, gallbladder, biliary ducts, or pancreas during follow-up (*n* = 499), and thus, 74,554 participants were left for analyses. Ethical approval was granted by the Regional Ethical Board at Karolinska Institutet.

### Dietary variables

Participants reported their usual FV consumption over the past year as part of a 96-item food frequency questionnaire. For each item, one of 8 frequency-of-consumption responses could be chosen (“never,” “1–3 times/month,” “1–2 times/week,” “3–4 times/week,” “5–6 times/week,” “1 time/day,” “2 times/day,” and “≥3 times/day”). There were 13 questions on vegetables (carrot, beetroot, lettuce, cabbage, cauliflower, broccoli, tomato, pepper, spinach, peas, onion, garlic, and other vegetables), five on fruit (orange, apple, banana, berry, and other fruit), and one on orange juice. In a validation study, which was conducted in a subsample of 129 women from the Swedish Mammography Cohort using a similar food frequency questionnaire (including 60 items), the Spearman correlation coefficients between the food frequency questionnaire and four 1-week diet records (completed 3–4 months apart) ranged from 0.4 to 0.7 (unpublished data). Frequency responses were converted to average consumption (servings/day), and the variable for total FV consumption was created by aggregating the consumption of all individual items [including that on orange juice, because the exposure–outcome association was highly similar with and without that item (data not shown)]. We also created variables for consumption of total fruit (including orange juice), total vegetables, citrus fruits (orange and orange juice), green leafy vegetables (lettuce and spinach), cruciferous vegetables (cabbage, cauliflower, and broccoli), and items high in vitamin C (cabbage, broccoli, pepper, orange, orange juice, berry, and other fruit).

Data on dietary co-variables (i.e., foods, beverages, and nutrients) were acquired from the food frequency questionnaire. Nutrients were adjusted for energy intake (1700 kcal/day in women and 2600 kcal/day in men) using the residual method [15].

### Other variables

The questionnaire contained questions on education, smoking status, physical activity, use of aspirin, height and weight, hyperlipidemia, and diabetes. Body mass index (BMI) was calculated as weight (kg) divided by height squared (m^2^). Additional data on the presence of hyperlipidemia and diabetes were obtained from the Swedish Patient Register and the Swedish Diabetes Register. The female questionnaire also contained questions on parity and use of oral contraceptives and postmenopausal hormones.

### Follow-up for cholecystectomy

We performed follow-up for cholecystectomy between January 1, 1998, and December 31, 2011, via linkage to the Swedish Patient Register (represented by codes JKA20 and JKA21 in the Swedish Classification of Operations and Major Procedures). Data on cholelithiasis and cholecystitis in relation to the surgical procedure were obtained from the same register (represented by codes K80 and K81 in the International Classification of Diseases-10). Data on cancer and death were acquired from the Swedish Cancer Registry and the Swedish Cause of Death Register, respectively.

### Statistical analyses

We used Cox regression models to obtain hazard ratios (HRs) according to sex-specific quartiles of FV consumption (with attained age during follow-up as time-axis) [16]. Entry time was defined as age at January 1, 1998, and exit time was defined as age at cholecystectomy, death, or December 31, 2011 (end of follow-up).

Potential confounders were selected on the basis of previous studies on the risk of cholecystectomy in the Swedish Mammography Cohort and the Cohort of Swedish Men [17, 18] and on the basis of other studies that have examined the association between FV consumption and risk of gallstone disease [7–14]. Included in the multivariable analyses were sex, education, smoking status, alcohol drinking, use of aspirin, energy intake, coffee consumption, and physical activity (measured as time spent walking; see Table 2, footnotes, for categorization). Adjusting for other aspects of physical activity (i.e., exercise, work occupation, and household work) had negligible influence on the results (data not shown). Further adjustment for (1) potential intermediates [i.e., BMI (<25, 25–29, ≥30 kg/m^2^), hyperlipidemia (no, yes), and diabetes (no, yes)], (2) nutrient intakes [i.e., sex-specific quartiles (g/day) of total fat, dietary fiber, carbohydrates, and protein, with the aim to examine the association between FV consumption and risk of cholecystectomy independent of these nutrients], and (3) female-specific factors [i.e., parity (nulliparous, parous) and use of oral contraceptives (never, ever) and postmenopausal hormones (premenopausal, never, ever)] did not change the results. Missing data on co-variables (see Table 1, footnotes) were handled using the missing indicator approach. Sensitivity analyses were performed using the complete-case approach, without any clear change in the results (data not shown).

**Table 1:** Age-standardized baseline characteristics by sex-specific quartiles of fruit and vegetable consumption

Characteristics^a^	Quartiles of consumption (servings/day) (median)							
	Men (*n* = 42,516)				Women (*n* = 32,038)			
	<2.4 (1.7)	2.4–3.5 (3.0)	3.6–5.1 (4.3)	>5.1 (6.5)	<3.3 (2.4)	3.3–4.7 (4.0)	4.8–6.6 (5.6)	>6.6 (8.3)
No. of participants	10,631	10,650	10,613	10,622	8013	8008	8009	8008
Age (years) (mean)	61.0	59.7	59.3	59.7	63.6	61.6	61.0	60.2
Education >12 years (%)	9.2	14.4	18.0	23.2	12.4	19.0	23.5	27.1
Current smoker (%)	33.7	24.4	21.4	18.3	27.7	19.8	17.0	14.0
BMI (kg/m^2^) (mean)	26.0	25.8	25.6	25.6	25.0	24.9	24.8	24.8
Physical activity >40 min of walking/day (%)	27.8	32.0	34.2	37.3	30.0	33.3	36.8	42.0
Use of aspirin (%)	35.7	38.2	38.0	38.0	50.2	51.9	51.4	50.8
History of diabetes (%)	10.4	9.7	8.5	9.9	4.3	4.2	4.1	4.7
History of hyperlipidemia (%)	16.6	16.5	17.0	16.2	8.6	8.2	8.9	9.1
Ever used oral contraceptives (%)	–	–	–	–	58.1	59.4	60.9	60.2
Parity (mean)	–	–	–	–	2.1	2.1	2.1	2.2
Ever used HRT (%)^b^	–	–	–	–	50.6	54.6	56.2	58.1
Daily intake (mean)								
Alcohol (g)^c^	15.4	15.0	15.0	14.9	6.4	6.7	6.7	6.9
Coffee (cups)	3.7	3.5	3.4	3.3	3.2	3.1	3.0	3.0
Energy (kcal)	2390	2586	2714	2992	1513	1680	1790	2026

*BMI* body mass index, *HRT* hormone replacement therapy

^a^Means and percentages were calculated for men and women with complete data. The percentage of missingness was 0.3 % for education, 1.5 % for smoking status, 3.5 % for BMI, 8.6 % for physical activity, 9.9 % for use of aspirin, 1.0 % for use of oral contraceptives, 5.8 % for use of HRT, 2.4 % for alcohol intake, and 4.9 % for coffee consumption

^b^Calculated for postmenopausal women

^c^Calculated for current drinkers

A test for proportionality of hazards according to quartiles of FV consumption was conducted with the Schoenfeld test [19]. In practice, this corresponds to a test for an interaction with age when attained age is the time-axis. In the case of formal evidence of non-proportionality, we allowed hazards to vary flexibly by including an interaction term between FV consumption (in quartiles) and attained age (as a continuous variable, modeled using 3-knot restricted cubic splines) [20]. We also tested for an interaction with sex (using the likelihood ratio test) and performed separate analyses of men and women as sensitivity analyses. Analyses were two-sided at the 5 % significance level and performed with Stata 12.1 (StataCorp, TX, USA).

## Results

A median of 4.1 daily servings of FVs were consumed at baseline (4.8 servings in women and 3.6 servings in men) including 1.4 servings of fruit (1.7 servings in women and 1.2 servings in men) and 2.5 servings of vegetables (2.9 servings in women and 2.2 servings in men). High consumers of FVs were less likely to be current smokers and more likely to be well educated, physically active, and users of postmenopausal hormones (Table 1). (See Electronic Supplementary Table S1 for baseline characteristics separated by fruit consumption and vegetable consumption.) During a total follow-up of 939,715 person-years (1998–2011: mean of 12.6 years and 25th percentile of 14.0 years), 2120 participants underwent a cholecystectomy (1120 women and 1000 men), of whom 2007 had a prior or concomitant diagnosis of cholelithiasis or cholecystitis.

In age- and sex-adjusted analyses, FV consumption was inversely associated with risk of cholecystectomy (Table 2). However, after adjustment for potential confounders, particularly for education, physical activity, and energy intake, the inverse association was attenuated. The multivariable-adjusted HR was 0.95 (95 % CI 0.83–1.08) for the highest compared with the lowest sex-specific quartile of FV consumption (Table 2). Further adjustment for potential intermediates, that is, BMI, hyperlipidemia, and diabetes (HR 0.95; 95 % CI 0.83–1.08), as well as for intakes of total fat, dietary fiber, carbohydrates, and protein (HR 0.94; 95 % CI 0.82–1.09), did not change the results. Null associations were also observed when various lag times between diet assessment and start of follow-up were applied [3 years (*n* = 72,262 including 1549 cases) to 5 years (*n* = 70,196 including 1200 cases), with the aim to reduce the possibility that FV consumption had been affected by latent symptoms of gallstones] (data not shown). Likewise, the risk of cholecystectomy was not associated with consumption of total fruit (HR 0.98; 95 % CI 0.86–1.13), total vegetables (HR 1.00; 95 % CI 0.87–1.14), citrus fruits (HR 0.95; 95 % CI 0.83–1.09), green leafy vegetables (HR 1.09; 95 % CI 0.94–1.26), cruciferous vegetables (HR 0.94; 95 % CI 0.83–1.07), or items high in vitamin C (HR 0.93; 95 % CI 0.80–1.07).

**Table 2:** Hazard ratios of cholecystectomy by sex-specific quartiles of fruit and vegetable consumption

	Quartile of consumption^a^				*P* for trend^b^
	1	2	3	4	
No. of participants	18,644	18,658	18,622	18,630	–
No. of cases/person-years	532/227,763	534/235,426	535/237,903	519/238,624	–
Hazard ratio (95 % CI)					
Age- and sex-adjusted	1.00 (ref)	0.94 (0.83–1.06)	0.92 (0.82–1.04)	0.88 (0.78–0.99)	0.036
Multivariable-adjusted^c^	1.00 (ref)	0.96 (0.85–1.09)	0.96 (0.85–1.09)	0.95 (0.83–1.08)	0.43

^a^See Table 1 for range (servings/day) of sex-specific quartiles of fruit and vegetable consumption in men and women

^b^Test for trend was calculated by modeling the sex-specific quartiles of FV consumption as a continuous variable

^c^Derived from a Cox regression model that was adjusted for attained age during follow-up (time-axis), sex, education (≤12, >12 years), smoking status (never, past, current), alcohol drinking [never, past, current in < or ≥ the sex-specific median intake (g/day)], physical activity (<20, 20–40, >40 min of walking/day, corresponding to approximate tertiles), use of aspirin (no, yes), energy intake [sex-specific quartiles (kcal/day)], and coffee consumption (<2, 2–3, 4–5, ≥6 cups/day)

**Fig. 1:**
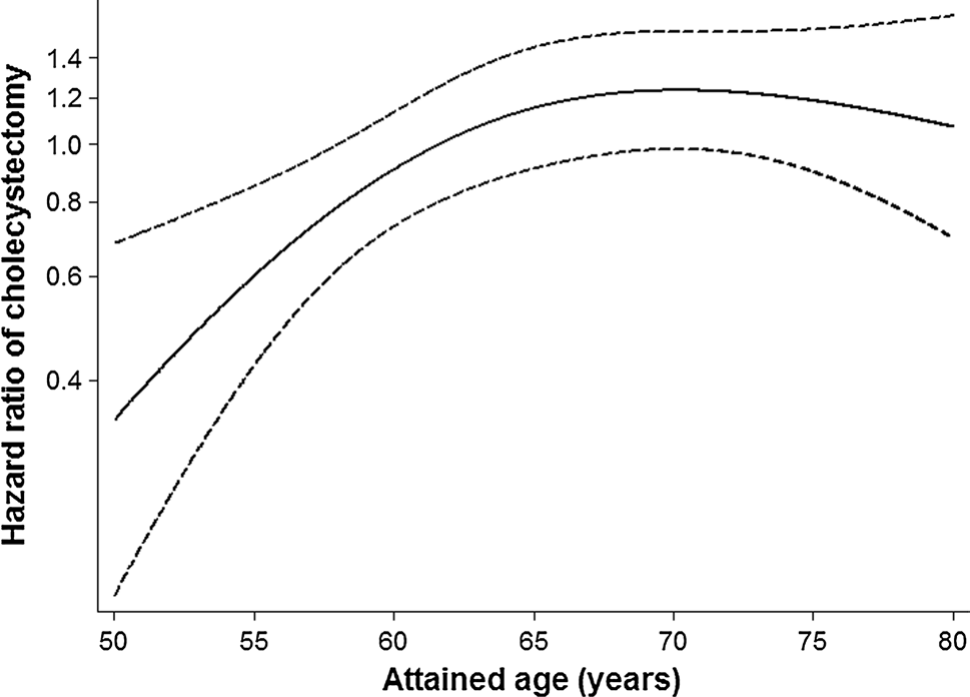
Time-varying hazard ratios of cholecystectomy for the highest compared with the lowest quartile of fruit and vegetable consumption in women, according to attained age during follow-up (*time-axis*). The figure illustrates time-varying hazard ratios and 95 % CI for the highest compared with the lowest quartile of fruit and vegetable consumption (*Y-axis*) according to attained age (*X-axis*). Data were derived from a Cox regression model that included an interaction term between fruit and vegetable consumption (*in quartiles*) and attained age (as a continuous variable, modeled using 3-knot restricted cubic splines). Estimates were adjusted for the same co-variables as those in Table 2

There was no evidence of interactions between FV consumption and age (*P* ≥ .25) or sex (*P* = .72) in relation to risk of cholecystectomy, with multivariable-adjusted HRs of 0.92 (95 % CI 0.76–1.10) and 0.97 (95 % CI 0.81–1.16) in men and women, respectively, for the highest compared with the lowest quartile of FV consumption. Further adjustment for parity and use of oral contraceptives and postmenopausal hormones resulted in a HR of 0.95 (95 % CI 0.80–1.14) in women. However, in contrast to the analyses pooled by sex, there was evidence of an interaction with age in the women who were high consumers of FVs (*P* = .010 for quartile 4 and *P* ≥ .51 for quartile 2 and 3). A high FV consumption was associated with decreased HRs of cholecystectomy for ages up to 60 years, with seemingly linear decreases in magnitude in that age range, whereas for ages of more than 60 years, there was no such association (Fig. 1). Fifty-two percentage of the women were aged 60 years or less at baseline (*n* = 16,575), who contributed to 26 % of the total number of person-years of follow-up (*n* = 106,560) and 34 % of the total number of cases of cholecystectomy (*n* = 382) before they turned 61 years. No evidence of an interaction with age was observed in men (*P* ≥ .23).

## Discussion

In this large prospective cohort study, we found evidence of an inverse association between FV consumption and risk of cholecystectomy in women, although the association was restricted to women aged 48–60 years. In contrast, there was no evidence of an association in men.

Our finding of a null association with FV consumption in the combined analysis of women and men is in agreement with the few (and small) studies that have (1) examined its association with gallstone disease and (2) included both women and men [10–14], with the exception of a Spanish case–control study in which gallstone patients consumed less fruit than did control subjects [8]. However, on closer scrutiny, those results were solely driven by the female cases, as the male cases had a higher mean fruit consumption than had the control subjects. In fact, the only significant findings reported in the literature have been in women [7–9]. As such, our finding of an inverse association with FV consumption in women aged 60 years or less also finds some support. In a small case–control study of Greek women (mean age among control subjects 54 years), gallstone patients consumed less vegetables than did control subjects [9]. An inverse association with fruit consumption was in turn observed in the small case–control study of Spanish women mentioned above (mean age among control subjects 64 years) [8]. Finally, an inverse association with FV consumption was observed in the NHS (mean age at the start of a 17-year follow-up period 50 years), the only large prospective cohort study to date [7]. It should be noted that most between-study comparisons are limited because of the mixture of study designs (i.e., cross-sectional, case–control, or cohort) and outcome definitions (i.e., presence of gallstones on ultrasound and/or occurrence of cholecystectomy).

Due to similarities in study design and outcome definition, a thorough comparison between the present study and the NHS is needed [7]. Among 77,090 women, followed up between 1984 and 2000, there was an inverse association between FV consumption and risk of cholecystectomy (*P*_trend_ < .0001). The multivariable-adjusted HR was 0.79 (95 % CI 0.73–0.87) for the highest compared with the lowest quintile of FV consumption—and, in contrast to our study, there was no evidence of an interaction with age (HR for <60 years 0.80 and HR for ≥60 years 0.78). Some differences between the two studies should also be noted, since they may affect generalizability and explain discrepancies in results. Firstly, and of importance regarding a potential interaction with age, the NHS had a younger age structure (aged 37–64 years at the start of a 17-year follow-up period) than had our cohort of women (aged 48–84 years at the start of a 14-year follow-up period). Secondly, adjustment for potential confounders had a minimal effect on the results in the NHS, which is a rather homogenous cohort of nurses, whereas the age- and sex-adjusted results in our population-based cohort were clearly attenuated in the multivariable-adjusted analyses. Finally, the crude incidence of cholecystectomy was very high in the NHS (6.2 cases per 1000 person-years), especially compared with that in our cohort of women (2.7 cases per 1000 person-years) as well as that in other European cohorts of women (2.8–3.4 cases per 1000 person-years) [21, 22].

Since gallstone disease is considerably more common in women than in men [23], which suggests sex differences in the etiology, it is plausible that the association with FV consumption might differ by sex. Higher levels of female sex hormones have been associated with an increased risk of gallstone disease, for example due to postmenopausal hormones [17, 21, 22, 24] (including in our data [17]) and multiple childbirths [25] [including in our data, with multivariable-adjusted HRs (95 % CI) of 1.37 (1.04–1.81), 1.43 (1.12–1.83), and 1.54 (1.20–1.98) for 1, 2, and ≥3 childbirths, respectively, compared with nulliparity]. Experimental studies have demonstrated that overexpression of progesterone receptors may lead to a slow intestinal transit [26, 27], while clinical studies have shown that women’s intestinal transits are slower than men’s, which seem to depend on the menstrual cycle [28]. A slow intestinal transit may, in turn, increase the risk of gallstone disease by promoting bacterial overgrowth and by increasing the intestinal absorption of cholesterol [29–31]. FVs are rich in dietary fiber, which shortens the intestinal transit [32] and has been inversely associated with risk of gallstone disease [33]. Thus, it might be that an inverse association between FV consumption and risk of gallstone disease depends on female sex hormones, as well as the level of these, which may explain our finding of a lack of association in men and of an interaction with age in women.

Some strengths of our study were its large size, inclusion of women and men, prospective assessment of diet, and ability to control for a number of potential confounders. Other strengths included the population-based design and an almost complete follow-up via linkage to national health registers.

The major limitation of the study was the reliance on self-reported diet, which, inevitably, must have led to some degree of misclassification of FV consumption. Also, since the exposure was only measured once at baseline, further misclassification might have occurred as a result of potential changes in FV consumption during follow-up. However, due to the study’s prospective design, such types of misclassification should have been unrelated to the future occurrence of cholecystectomy. Because of the age structure of the participants (aged 45–83 years at baseline), we could not determine how the risk of cholecystectomy is influenced by FV consumption earlier in life. Furthermore, we used gallstone disease that required cholecystectomy as the outcome of interest. Our results may, therefore, not be generalizable to patients with asymptomatic gallstone disease or to those with conservatively treated gallstone disease. However, apart from the fact that the probability of false-positive cases should have been low, we believe that patients treated with cholecystectomy represent the most clinically relevant fraction of gallstone disease. Since the cohorts consisted almost exclusively of non-Hispanic whites, the generalizability to populations of other ethnicities is also expected to be limited. On the other hand, the ethnically homogenous population should have kept the influence of confounding by genetic factors to a minimum. Finally, as in all observational studies, we cannot rule out the possibility of residual and/or unmeasured confounding (for example due to rapid weight loss and dieting [34], two co-variables for which we had no satisfactory data).

In conclusion, this prospective cohort study supports an inverse association between FV consumption and risk cholecystectomy in women, although the association was restricted to women aged 48–60 years. In contrast, the study does not support an association in men. Given the sparse epidemiologic data on the association between FV consumption and gallstone disease, particularly from large prospective cohort studies, further observational studies are required in both women and men.

## Electronic supplementary material

Below is the link to the electronic supplementary material.

Supplementary material 1 (DOCX 20 kb)
