# Supplementary material for: Fruit and vegetable consumption and risk of cholecystectomy: a prospective cohort study of women and men
Source: Eur J Nutr. 2016 Aug 20;57(1):75–81. doi: 10.1007/s00394-016-1298-6 (PMC5847035; doi:10.1007/s00394-016-1298-6)
Supplement: Supplementary file 1 — Supplementary material 1 (DOCX 20 kb) [file 394_2016_1298_MOESM1_ESM.docx]

**Fruit and vegetable consumption and risk of cholecystectomy: a prospective cohort study of women and men**

European Journal of Nutrition

Caroline Nordenvall,* Viktor Oskarsson,^†^ and Alicja Wolk^†^

^*^Department of Molecular Medicine and Surgery, Karolinska Institutet, and Center for Digestive Diseases, Karolinska University Hospital, SE-171 76 Stockholm, Sweden

^†^Institute of Environmental Medicine, Karolinska Institutet, SE-171 77 Stockholm, Sweden

Correspondence: Caroline Nordenvall (e-mail: caroline.nordenvall@ki.se)

**Table S1.** Age-standardized baseline characteristics by sex-specific quartiles of fruit consumption and vegetable consumption

|  | | **Quartiles of consumption (servings/day)** | | | | | | | | |
| --- | --- | --- | --- | --- | --- | --- | --- | --- | --- | --- |
|  | | **Fruit^a^** | | | |  | **Vegetables^b^** | | | |
| **Characteristics^c^** | | **1** | **2** | **3** | **4** |  | **1** | **2** | **3** | **4** |
| No. of participants | | 18,864 | 18,656 | 18,547 | 18,487 |  | 18,771 | 18,606 | 18,540 | 18,637 |
| Age (years) (mean) | | 60.8 | 60.5 | 60.4 | 60.9 |  | 62.6 | 60.6 | 59.8 | 59.5 |
| Male sex (%) | | 57.1 | 57.1 | 56.9 | 57.0 |  | 58.3 | 56.7 | 56.9 | 57.0 |
| Education >12 years (%) | | 13.0 | 16.3 | 20.4 | 22.9 |  | 10.5 | 16.1 | 20.6 | 24.5 |
| Current smoker (%) | | 31.8 | 22.5 | 18.0 | 16.0 |  | 28.6 | 22.1 | 19.7 | 18.5 |
| BMI (kg/m^2^) (mean) | | 25.5 | 25.5 | 25.3 | 25.2 |  | 25.6 | 25.3 | 25.3 | 25.3 |
| Physical activity >40 min of walking/day (%) | | 29.0 | 32.9 | 34.4 | 39.2 |  | 29.5 | 32.5 | 35.4 | 38.4 |
| Use of aspirin (%) | | 42.7 | 43.7 | 43.3 | 42.4 |  | 41.2 | 43.7 | 43.6 | 43.8 |
| History of diabetes (%) | | 8.1 | 7.2 | 7.1 | 6.7 |  | 7.5 | 6.7 | 6.8 | 8.0 |
| History of hyperlipidemia (%) | | 13.0 | 13.5 | 13.1 | 12.9 |  | 13.2 | 12.6 | 13.4 | 13.4 |
| Ever used oral contraceptives (%)^d^ | | 59.7 | 59.4 | 60.5 | 59.0 |  | 57.3 | 59.2 | 60.8 | 61.0 |
| Parity (mean)^d^ | | 2.2 | 2.1 | 2.1 | 2.1 |  | 2.1 | 2.1 | 2.1 | 2.2 |
| Ever used HRT (%)^e^ | | 51.8 | 55.6 | 55.3 | 56.6 |  | 50.4 | 54.4 | 56.4 | 58.2 |
| Daily intake (mean) | |  |  |  |  |  |  |  |  |  |
|  | Alcohol (g)^f^ | 13.4 | 11.6 | 11.3 | 11.0 |  | 11.6 | 11.4 | 11.8 | 12.5 |
|  | Coffee (cups) | 3.5 | 3.3 | 3.2 | 3.2 |  | 3.5 | 3.3 | 3.3 | 3.2 |
|  | Energy (kcal) | 2038 | 2198 | 2326 | 2546 |  | 2080 | 2213 | 2310 | 2517 |

Abbreviations: BMI; body mass index; HRT, hormone replacement therapy.

^a^Range of consumption in servings/day for men: first quartile (<0.7), second quartile (0.7–1.1), third quartile (1.2–1.9), and fourth quartile (>1.9); and for women: first quartile (<1.1), second quartile (1.1–1.6), third quartile (1.7–2.6), and fourth quartile (>2.6).

^b^Range of consumption in servings/day for men: first quartile (<1.4), second quartile (1.4–2.1), third quartile (2.2–3.2), and fourth quartile (>3.2); and for women: first quartile (<1.9), second quartile (1.9–2.8), third quartile (2.9–4.0), and fourth quartile (>4.0).

^c^Means and percentages were calculated for men and women with complete data (see Table 1 for the individual percentages of missing data).

^d^Calculated for women.

^e^Calculated for postmenopausal women.

^f^Calculated for current drinkers.
